# Applications of machine learning and deep learning in musculoskeletal medicine: a narrative review

**DOI:** 10.1186/s40001-025-02511-9

**Published:** 2025-05-15

**Authors:** Martina Feierabend, Julius Michael Wolfgart, Maximilian Praster, Marina Danalache, Filippo Migliorini, Ulf Krister Hofmann

**Affiliations:** 1 Metabolic Reconstruction and Flux Modelling, University of Cologne, Zülpicher Str. 47b, 50674 Cologne, Germany; 2 Department of Orthopaedic, Trauma, and Reconstructive Surgery, RWTH University Hospital, 52074 Aachen, Germany; 3 Department of Orthopaedic, Trauma, and Reconstructive Surgery, Division of Arthroplasty and Tumour Surgery, RWTH University Hospital, 52074 Aachen, Germany; 4 Teaching and Research Area Experimental Orthopaedics and Trauma Surgery, RWTH University Hospital, 52074 Aachen, Germany; 5 Department of Orthopaedic Surgery, University Hospital Tübingen, Hoppe-Seyler Straße 3, 72076 Tübingen, Germany; 6 Department of Orthopaedic and Trauma Surgery, Academic Hospital of Bolzano (SABES-ASDAA), Teaching Hospital of the Paracelsus Medical University, 39100 Bolzano, Italy

**Keywords:** Artificial intelligence, Machine learning, Supervised learning, Unsupervised learning, Reinforcement learning, Orthopaedics, Traumatology

## Abstract

Artificial intelligence (AI), with its technologies such as machine perception, robotics, natural language processing, expert systems, and machine learning (ML) with its subset deep learning, have transformed patient care and administration in all fields of modern medicine. For many clinicians, however, the nature, scope, and resulting possibilities of ML and deep learning might not yet be fully clear. This narrative review provides an overview of the application of ML and deep learning in musculoskeletal medicine. It first introduces the concept of AI and machine learning and its associated fields. Different machine concepts such as supervised, unsupervised and reinforcement learning will then be presented with current applications and clinical perspective. Finally deep learning applications will be discussed. With significant improvements over the last decade, ML and its subset deep learning today offer potent tools for numerous applications to implement in clinical practice. While initial setup costs are high, these investments can reduce workload and cost globally. At the same time, many challenges remain, such as standardisation in data labelling and often insufficient validity of the obtained results. In addition, legal aspects still will have to be clarified. Until good analyses and predictions are obtained by an ML tool, patience in training and suitable data sets are required. Awareness of the strengths of ML and the limitations that lie within it will help put this technique to good use.

## Introduction

**Fig. 1:**
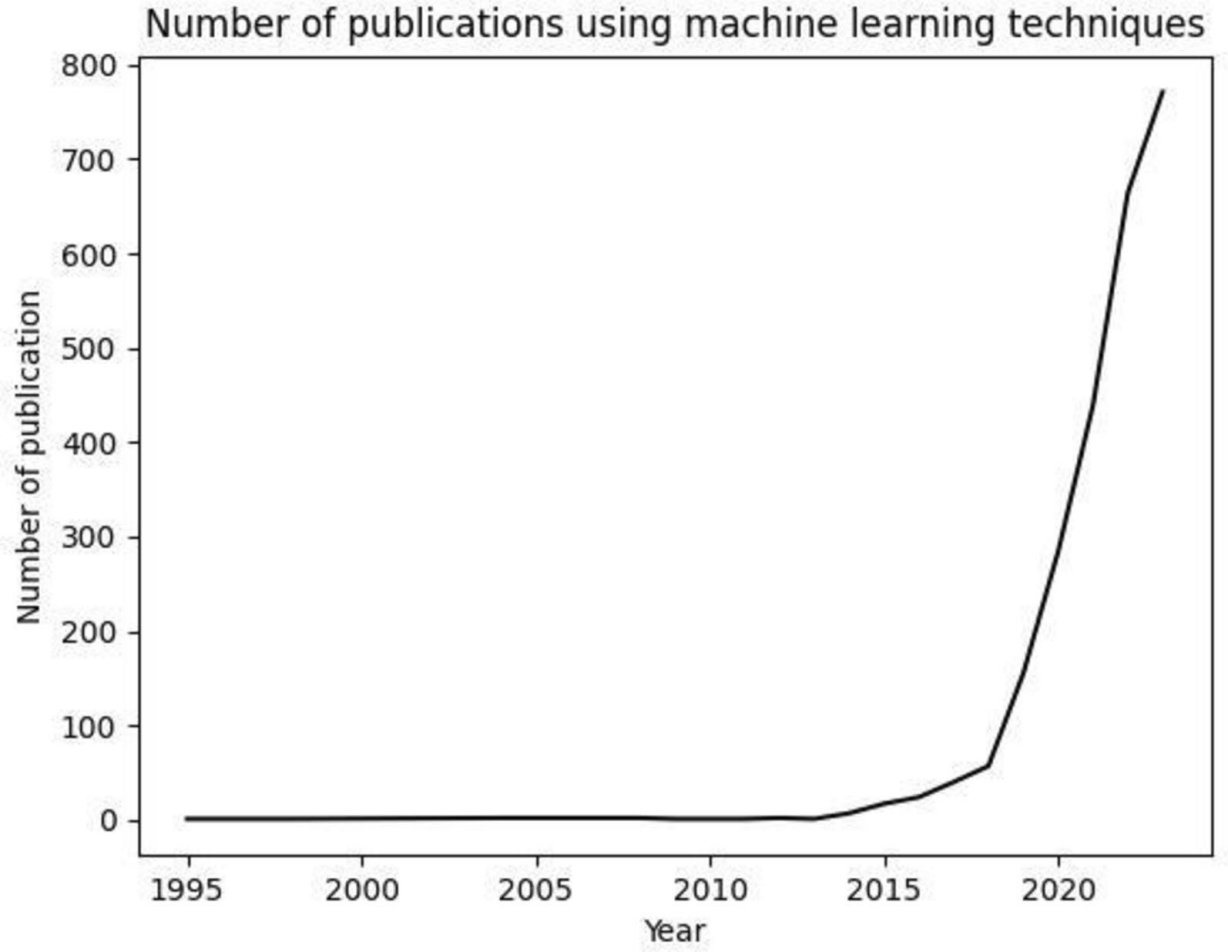
Number of publications using machine learning in orthopaedics or traumatology has increased exponentially in the last 10 years. A PubMed search was conducted using the search terms “orthopaedics,” “traumatology,” and “machine learning.” The time range includes publications until 2022. While these techniques were hardly in use until 2015, the number of new publications has risen exponentially in the last decade and continues to do so

Artificial intelligence (AI) and its technologies such as machine learning (ML) have the potential to transform many aspects of patient care and patient administration in all fields of health care [23]. Over the last few years, ML techniques have become ubiquitous in more and more research fields outside computer science. This observation is closely associated with progress within computer science, mainly larger computational power and memory capacity, allowing ML to regain and even increase its popularity [67, 73]. In addition, the availability of easy-to-use open-source software libraries (e.g., R, cran.r-project.org, Python TensorFlow, tensorflow.org) has eased the burden of researchers with noncomputational backgrounds to include these techniques for their research questions. With these advances, ML models could reduce their error rates in object recognition by almost half in the past few years [1, 59], making thus ML also more attractive for the medical field. Indeed, ML techniques were hardly used in orthopaedics and traumatology until 2015. Since then, the number of new publications has risen exponentially and continues to do so (Fig. 1). Potential benefits of including ML in a clinical setting could be better patient care [39, 82], aided decision processes for surgeons, [100] or better clinical management and resource allocation [39], to name just a few. High amounts of digitally collected patients ‘ data in large databases and medical registries provide ideal working conditions to apply ML techniques to various healthcare questions [30]. As such, the field of orthopaedics is increasingly suitable for the application of ML as the amount of available data in already existing orthopaedic registries [e.g., Network of Orthopaedic Registries of Europe [105] (Zaffagnini et al.); AAOS Registry Program; International Society on Arthroplasty Registers, ISAR] belongs to the largest gathered in healthcare. Current research on machine learning using clinical data is, however, still faced with the dilemma that available data sets are often unstructured. To make use of these data, they first have to be annotated which, to date, usually still involves human labour.

However, for many clinicians working with and on musculoskeletal medicine, the nature of ML and deep learning, its scope, and future possibilities might not be fully clear.

This narrative provides an overview of the theoretical constructs and latest developments in ML and deep learning, focusing on musculoskeletal medicine. It first introduces the concept of AI and machine learning and its associated fields. Different machine concepts such as supervised, unsupervised and reinforcement learning will then be presented. The review will then delve into deep learning and discuss potential developments in the field.

### Definition of artificial intelligence

AI was initially described as making a machine behave in ways that would be called intelligent if a human were so behaving [68, 87]. The concept of “artificial” intelligence is thereby contrasted with the “natural” intelligence of biological life forms.

AI is a broad interdisciplinary approach encompassing several subdisciplines such as ML with its subset deep learning, machine perception, robotics, natural language processing, or expert systems [87]. AI was developed to understand, model, and create intelligence of various forms [31]. Its different concepts and approaches are based on mathematical calculations to generate a probabilistic representation of uncertainty, which is then used to make predictions about future data or to come to decisions based on the given predictions [36]. This is especially advantageous in a context, where the abundance of data is too complex to handle with conventional means. In such a context, predictive models allow us to ascertain the association between variables (e.g., patient characteristics) and events (e.g., surgical outcome) useful for decision-making, surgical planning, or postoperative rehabilitation protocols [7, 8, 90]. Most forms of AI can process not only structured but also semi- or unstructured data. Thus, human intelligence is replaced by data-driven algorithms integrated into a dynamic computing environment. These computational systems, albeit still very limited in their actual cognitive dimension, are intended to possibly in the future also allow these systems to learn, reason, consider, reflect, and perform cognitive functions typically associated with human cognition [7, 44, 82]. Presently, numerous AI applications have been established that are, however, still largely task-specific, and in many cases at an experimental stage. One example for an already implemented task specific application is the detection of landmarks on X-rays and a computer-based planning of component positioning in total joint arthroplasty [72]. One rather general problem with the implementation of such models is the heterogeneity of data in medicine. To be able to design and train a model than can reliably predict outcome or recommend medical treatments, large data sets need to be available that include also long-term follow-up data with subjective patient reported outcome measures and at the same time objective and quantifiable measures.

#### Machine learning

The development of AI applications is usually triggered or accompanied by the implementation or at least significant improvements of other computer-related techniques that allow AI to receive input from the environment. In the last decade, for example, great advances have been made in the field of computer vision. Computer vision based on AI can now directly extract information from images and videos. AI-based tools can thus be trained to interpret radiographs. In the musculoskeletal field, radiographic imaging is, for example, essential to diagnose and manage fractures and trauma cases in emergency rooms as it provides a quick and cost-effective method to identify bone pathologies. Despite its widespread use, however, radiographic misdiagnoses remain common in the fast-paced, high-pressure ER environment. These errors can lead to delays in treatment, inappropriate management, and long-term complications. In this context, integrating properly trained artificial intelligence models can help reduce misdiagnoses, improve diagnostic accuracy, and ultimately enhance patient outcomes [77]. In a review article by Oeding and colleagues from 2024, for example, the majority of AI models demonstrated comparable or even better performance compared with human experts in detecting scaphoid and distal radius fractures [78]. In addition, in an elective setting, the use of ML techniques is increasingly helpful, as it has been shown to be able to give estimates on predicted disease progression of osteoarthritis and treatment outcomes [60]. Moreover, AI can be trained to screen for implant loosening on radiographs [54], measure knee alignment [93], and use these data to evaluate the radiological result of a performed total knee replacement [11].

#### Machine perception

Computer vision can also be used in the application of augmented reality. Augmented reality is a technique that provides the user with additional visual, auditory, haptic, somatosensory, or olfactory input [17]. In medicine, the application of augmented reality has been observed to lower the user's cognitive burden. In pre-clinical cadaveric and sawbones models, augmented reality could also reduce operative time and radiation exposure while improving surgical precision (reviewed by [34]). An orthopaedic application related to computer vision is its use in total knee arthroplasty (TKA). When exposed to adequate radiographs, AI can help with the preoperative planning of the implant [6, 60].

#### Robotics

When, next to the radiographic data, providing an AI system with information on flexion/extension gaps or when measuring patella tracking, AI helps to optimise the intraoperative decision-making algorithm, resource allocation, implant selection and implant positioning [6, 18, 62, 81]. The two primary clinical applications here are TKA navigation (e.g., OrthoPilot^®^ from Aesculap^®^) or TKA-robotics. TKA-robotics are available both as semi-active (e.g., MAKO™ from Stryker™; CORI from Smith&Nephew; OMNIBotics knee system from OMNIlife Science) or active systems (e.g., VELYS™ from DePuy Synthes; TSolution One from Think Surgical; ROSA Knee robotic system from Zimmer Biomet). For these navigation or robotic techniques, data are collected on the geometry of the bone surface and the movement of the extremity. Computational algorithms calculate implant alignment and soft tissue balancing based on this input.

#### Natural language processing

At the interface of the computational AI system and its perception of the environment is natural language processing (NLP). This refers to computational techniques used to extract meaning from humans' written or spoken language. As such, everyday linguistic tasks, such as describing language or finding a semantic context, are core elements of NLP. NLP basic algorithms usually break down a sentence into its essential compounds, such as words, and count the occurrence of each of them in a sentence. A more complex task in NLP would be to reduce disambiguation in the semantic context of words (e.g., the"surgical instrument"versus the"musical instrument"). Until now, NLP in orthopaedics has mainly been used for the automated recognition of dictated doctors’ reports, for example, from the consultation or the operation theatre [74]. Other applications, especially in the field of orthopaedic or trauma research, are the analysis of patient-reported outcome measures [38], the study of medical reports, such as radiological reports (e.g., evaluation of the presence of periprosthetic femur fractures) [95], or, e.g., the identification of common elements in reports (e.g., certain infections following a surgery, reviewed by [101]).

#### Expert systems

Expert systems are computer systems that simulate the decision-making capabilities of a human expert [80]. These systems are designed to solve complex problems by reasoning through existing bodies of knowledge. One subcategory is medical expert systems. These systems can capture domain knowledge from existing literature and human experts and offer justified diagnostic or therapeutic recommendations [88]. Creating expert systems is done the following way: first, a knowledge base has to be obtained on the investigated issue (e.g., sports trauma of the knee; hospital-acquired respiratory tract infection). This is done by thorough literature research and usually investigation of experts. Then, a reasoning engine is created that emulates an intelligent expert system diagnosis, which allows the user to quickly find diseases, diagnose injuries and get the best rehabilitation [15]. Medical expert systems are already in use, e.g., for classifying medical errors [55]. A typical „dialogue “ between medical staff and the medical expert system is described in Table 1.

**Table 1:** Typical"use-case"scenario for expert systems

Is the patient female or male?	Male
How old is the patient?	43
Did the patient stay overnight?	Yes
How many days?	7
State the number of medical staff that had contact with the patient.	6
Did the patient come into contact with staff through the following: medical devices, food, dispensations, etc.?	Yes
Does the hospital have a hand-cleansing protocol for staff?	No
The patient is likely to have contracted a hospital-acquired infection given the absence of a hand-cleansing protocol. This suggested outcome has a 75% certainty based on the following elements: - patient overnight - patient exposed to more than 5 medical staff members - no hand-cleansing protocol at hospital	

In this example, a patient has been admitted to the hospital with complications arising from influenza. After an appropriate recovery time, however, no physical improvement was apparent (permission from [55]

Despite numerous implementations of AI in orthopaedics, its application has several limitations in daily clinical practice. Understanding the concepts of AI will help to also better understand its limitations and imagine its potential. One subdiscipline of AI is ML with its subset deep learning, which will now be further elaborated upon.

### Machine learning and deep learning

**Fig. 2:**
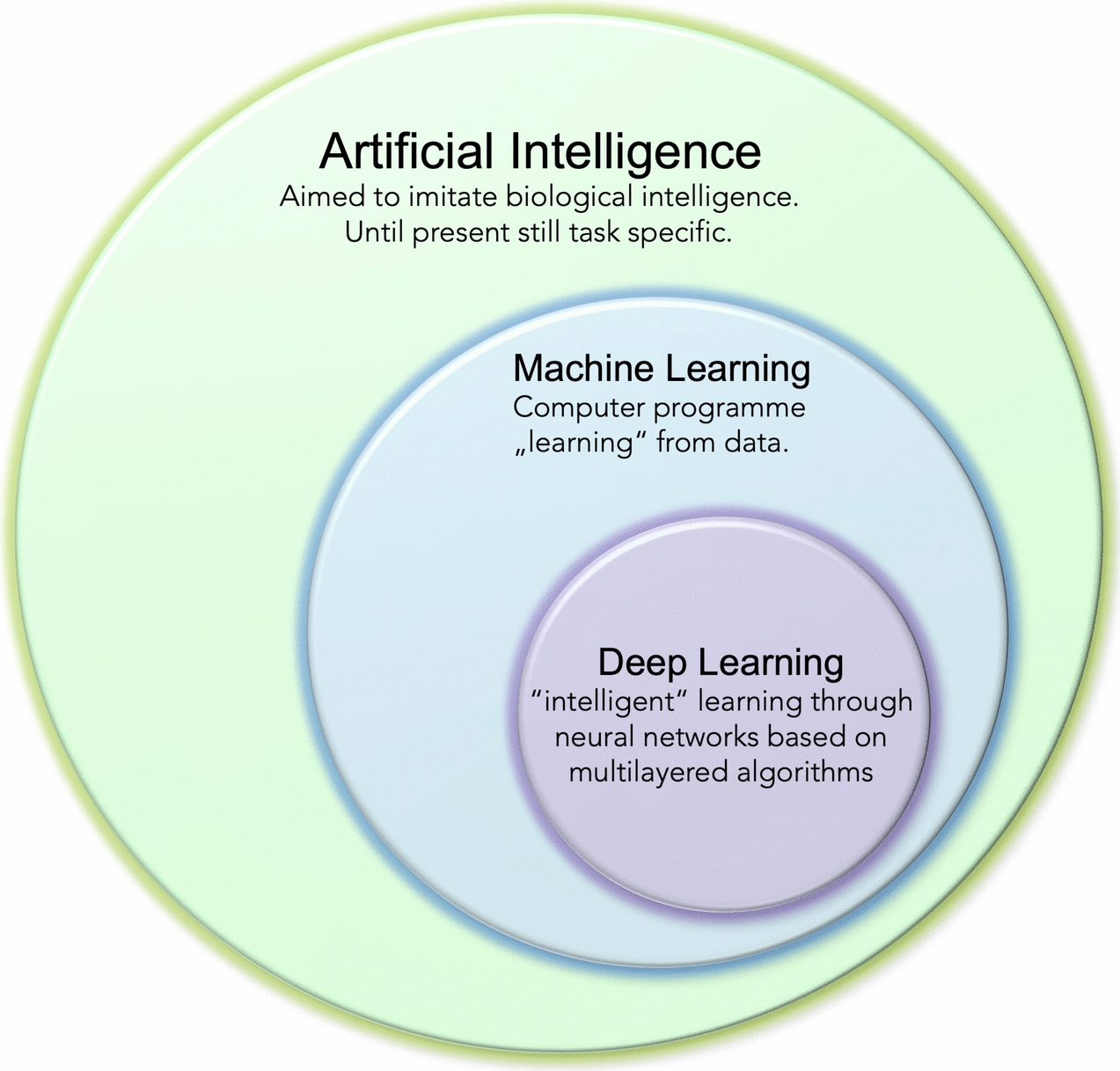
Within the concept of artificial intelligence, deep learning forms the"intelligent"subset of machine learning

**Fig. 3:**
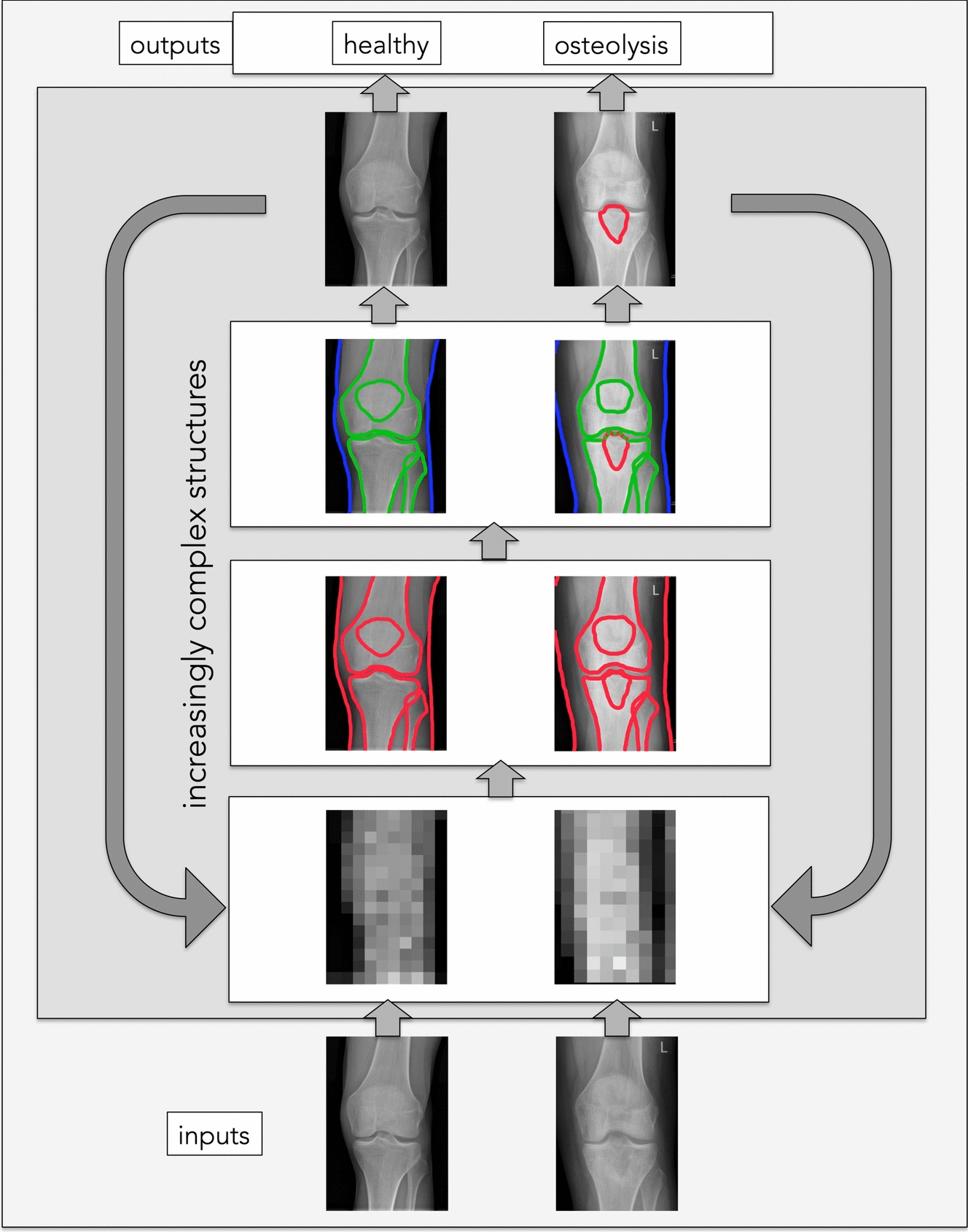
Machine learning model to detect osteolysis in a plain knee radiograph. Labelled input radiographs of healthy and pathological knees are given to the system. The training model then decomposes these images into grey value pixels. The model defines edges at areas of transition from higher to lower grey values. These edges are then aligned with the already-learned anatomy of a healthy knee radiograph. This feature extraction process involves identifying and capturing essential healthy and pathological knee characteristics. Aberrant lines are finally marked and labelled as pathologic. For the model creation, this process is repeatedly iterated to improve the diagnostic value of the model further

While AI is generally based on the idea that a machine can imitate human intelligence (i.e., to solve complex problems based on logic or decision-making trees), ML is explicitly task-specific and focuses on the learning process for specific tasks only. The learning process thereby serves solely the purpose of improving the results obtained for the designed task. ML is based on algorithms at the intersection of statistics, computer science, and AI. Since it lacks the"intelligence"aspect, it can only process structured and semi-structured data (except deep learning, see below) (Fig. 2). ML focuses on two closely connected aspects: first, on creating computationally based models that automatically improve through training. Second, ML also addresses the underlying statistical and computational laws which govern learning systems [46]. On a comprehensive level, a learning system can be defined as the query of improving certain performance measures when executing a specific task [46]. An example of how ML can help in orthopaedics to recognise a pathological condition on an X-ray is provided in Fig. 3.

**Fig. 4:**
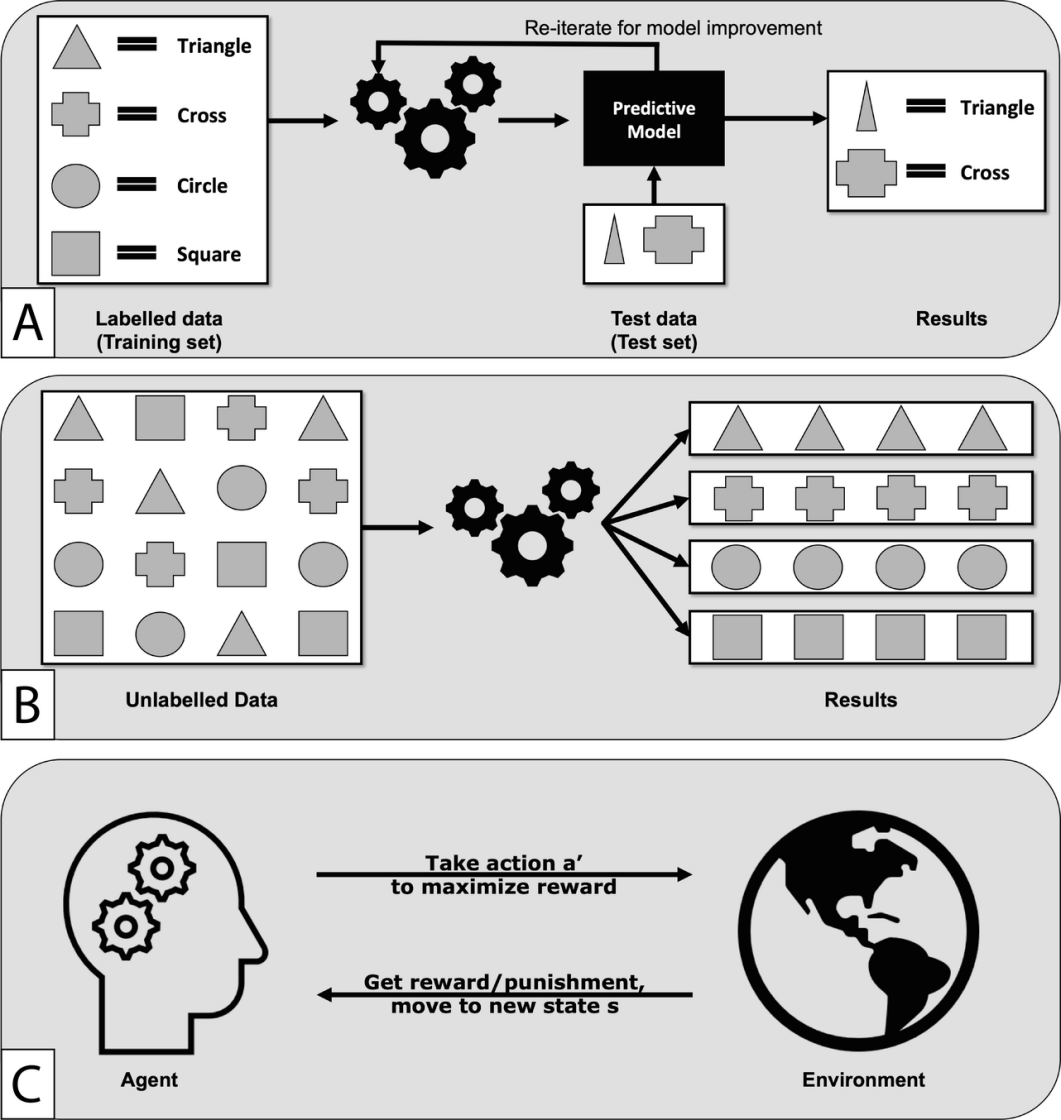
Three most common machine learning (ML) techniques. A machine learning model can be thought of as a complex web of interconnected nodes. Setting up an ML model involves two different kinds of data types: in the first step, training data are used to train the model. Once the model is set up in terms of its internal parameters, an unknown test dataset is used in a second step to validate the model. Finally, the model is used on new data. **A** Supervised learning problems can be sub-grouped into classification and regression techniques. In supervised learning, labelled data are used to train the model. This means that labelled input data are associated with a known outcome. The model is then trained on these data by an iterative process until fine-tuning of the model has been achieved. The model thus learns which features define the input data and how to identify them. This is done by applying weights, which represent numerical values assigned to connection nodes of the model. Weights determine the strength of these individual connections in the web of interconnected nodes and as such how strongly the output of a node influences another node’s input. Predictions made by supervised models can either be discrete or continuous. A model that produces discrete output data is a classification model (e.g., the result: tumour malignant or benign), and one that produces continuous output data is a regression model (e.g., the tolerable dose of a certain medication). **B** Unsupervised learning is used, e.g., clustering. Here, raw unlabelled data objects (on the left side) are provided as input. Training the model is also an iterative process. The results of unsupervised learning are often different clusters (as shown here with the non-overlapping geometrical shapes on the right). Clustering algorithms are used to assort the given data into groups that share common structures or patterns. **C** Reinforcement learning differs from supervised and unsupervised learning. In reinforcement learning, the model learns by the interactions between a decision maker/agent and its surrounding environment. The decision maker/agent selects an action according to its policy. Depending on the nature of the change in the environment, this action can be positive ("reward") which would reinforce the previous behaviour of the model, or negative ("punishment"). The goal of the model is to maximise its rewards

The three most widely used ML methods are supervised, unsupervised, and reinforcement learning (Fig. 4) [46]. Hybrid methods such as semi-supervised learning or multi-instance learning additionally exist. However, these will not be separately covered in this review.

#### Supervised learning

The idea of supervised learning algorithms (Fig. 4A) is that they create an ML model based on labelled data to generalise as accurately as possible [104]. The model is then trained to make accurate predictions on unknown data with the same characteristics as the labelled data. The workflow of supervised learning algorithms is as follows: a large set of training data of the form *[(*× *1, y1), …, (xn, yn)]* is given. This training set could, e.g., take the form of *[(femur1, bone), (radius1, bone), (Achilles ‘ tendon1, tendon), (patellar tendon1, tendon),…].* The training set is composed of a sample of independent and identically distributed pairs. During training, the machine is shown an image and produces an output in the form of a vector of scores, one for each category [59]. Then, the learning algorithm tries to find an objective function *g* from a space of possible functions *G*. This function *g i*s chosen to map the input and output data best. In other words, the error (or distance) between the output data and the input data is tried to get minimised. This is done by adjusting internal parameters to reduce the error [59]. To test if a generalisation of the model is valid, the performance of the model after training is measured on a different set of examples that the model has never seen during training [59]. Given its nature, supervised learning is most commonly used in situations involving regression or classification problems [91]. Common algorithmic methods to map supervised learning are decision trees, decision forests, logistic regression, support vector machines, neural networks and Bayesian classifiers [94]. Supervised learning requires labelled data, as is the case, for example, in orthopaedic or trauma registries: osteoporotic fractures of the pelvis, for example, have often several occult fracture sites which are difficult to detect but which have implications on the following treatment. Radiologic evaluation of periprosthetic infections of the hip or knee also remains a challenge for the clinician [40]. Connecting classifying data of these conditions with the original radiologic images might enable a neural network to evaluate these images independently. Uploaded original radiographs in registries is, however, still uncommon. For the German Arthroplasty Registry such a module is presently being discussed. Further already published applications of ML in orthopaedics are, for example, preoperative surgical assessment to identify optimal sagittal implant position in TKA [29], prediction of revision surgery after primary hip arthroscopy [66], prediction of surgery outcome such as survival rates in patients after treatment for chondrosarcoma [10], outcome after surgery of long bone metastasis [43], prediction of length of stay before primary TKA [45,76] or hip fracture [48], identification of patients at risk for prolonged opioid use after knee arthroscopy [63]. Already tested imaging applications are, for example, dual X-ray absorptiometry to detect hip fractures [57], CT scanning to detect lumbar osteoporosis [75], or relapse in rheumatoid arthritis patients using data on ultrasound examination [67].

#### Unsupervised learning

In certain situations, for example, when trying to discover structural properties in unlabelled data, a different method is usually applied, termed unsupervised learning (Fig. 4B). Unsupervised learning algorithms aims to find naturally occurring patterns or groupings within the data without any input from the user [91]. These structural properties can be algebraic, combinatorial or probabilistic [46]. Unsupervised learning methods allow compression of the information in a data set into fewer features, reducing the dimensionality of data [24, 46, 91]. The exploratory nature of unsupervised learning techniques is beneficial for identifying patterns and structures in high-dimensional data or high-dimensional problems [24]. Standard dimension reduction methods include principal component analysis, manifold learning, autoencoders, and factor analysis. These methods make different assumptions concerning the underlying manifold [46]. Clustering techniques are another example of unsupervised learning algorithms. Clustering methods usually calculate similarity and then use this similarity to group objects into clusters which are not known in advance. The clustering output is only helpful if the clusters correspond to the data, e.g., biologically relevant features that were not used to define the grouping. As such, external information is needed to judge the validity of clusters [3]. Both the dimension reduction and the clustering methods are preeminent in terms of their computational complexity, given that the goal is to exploit massive data sets when leaving out labelled data [46]. Recently, a classification tool for scoliosis using non-invasive surface measurements without prior knowledge of radiographic data was trained by unsupervised learning [20]. Other recent applications created by unsupervised learning are the identification of subgroups of patients at high, average or low fracture risks [56], the identification of divergent movement patterns that discriminate low back pain patients from healthy controls [51], the identification of vulnerable subpopulations among patients undergoing TKA or total hip arthroplasty patients based on only preoperative blood sample analysis [85], identifying patient clusters to predict quality of life after TKA [41].

#### Reinforcement learning

Reinforcement learning differs from the two forms of ML presented above. The training data in reinforcement learning are assumed to only indicate whether an action is correct or incorrect instead of displaying the proper output for a given input. In other words, reinforcement learning is a goal-directed learning technique. Learning occurs by interacting with the surrounding environments and observing status changes [19, 22, 46] (Fig. 4C). A typical and illustrative reinforcement learning scenario would be identifying the best possible racing line for a car in a computer game. The algorithm starts with random courses plotted for the vehicle. Each time in the iterative process, the algorithm exceeds its results from the previous random course during a predefined section of the race track, a reward is allocated to the programme. In case the performance is worse, a punishment is assigned. A formal description of reinforcement learning would thus be as follows: a problem is defined as consisting of a set of states in which the learning agent might find itself and a set of actions the agent can take. This setting then includes a transition function that describes how the environment will respond to the agent’s actions and a reward function that defines how good (or bad) observed events are. The reinforcement learning algorithms improve through the history of sequences of interaction (called histories) between the decision maker and their environment.

In a clinical setting, reinforcement learning algorithms have been tried, for example, to optimise sequences of decisions for long-term outcomes. Faced with a patient with sepsis, for example, the doctor in intensive care must decide if and when to initiate and adjust treatments, such as antibiotics, intravenous fluids, vasopressor agents, and mechanical ventilation. Each choice affects the patient’s survival at the end of the hospital stay and the patient’s quality of life upon recovery [37]. To perform sequential decision-making, such as for sepsis management, treatment-effect estimation must be solved at a grand scale and include numerous variable parameters. Reinforcement learning allows to take action in response to the changing environment and it can also include individual aspects of the patient [107].

#### Neural networks and deep learning

Deep learning is the subsection of machine learning based on artificial neural networks. As in other ML applications, these networks consist of an input layer, where data are entered and an output layer where results are obtained. In contrast to conventional ML, in"intelligent"deep learning, multiple such layers are superimposed, containing simple but non-linear modules. Each layer of interconnected nodes transforms the data from the previous layer into a representation at a higher, slightly more abstract level, leading to very complex functions [59].

**Fig. 5:**
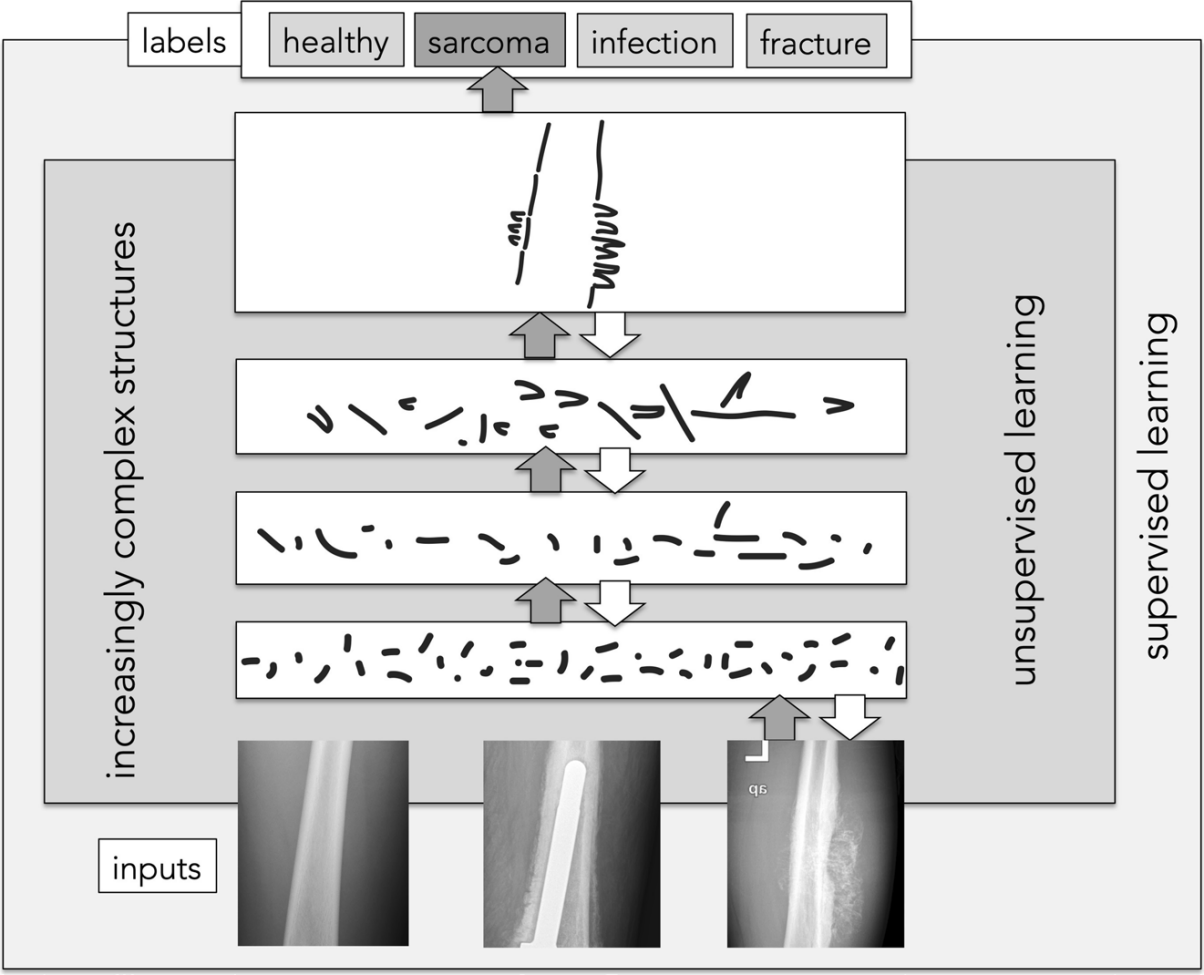
Exemplary neural-network architecture to detect sarcoma in a conventional knee radiograph. Adapted from Schulz et Behnke [89] with permission. In this example, four layers are superimposed: 1. The computer identifies pixels of light and dark. 2. The computer learns to identify edges and simple shapes. 3. The computer learns to identify more complex shapes and objects and integrates them into the notion of a bone radiograph. 4. The computer learns which shapes and objects can be used to identify a sarcoma in a human bone radiograph

Moreover, deep learning uses built-in algorithms, which modulate the programme to adapt its internal parameters to compute the representation in each layer from the representation in the previous layer. The multiple processing layers combined with their adaptive and recursive nature thus allow the programme to learn representations of data with various levels of abstraction through iterative adjustment [59] (Fig. 5), making deep learning so powerful. Moreover, the more data you feed in, the better the programme gets [14]. With the large amount of digital data that is now increasingly available, deep learning models are also increasing. In contrast to other ML techniques, deep learning is also capable of processing unstructured data without pre-processing usually required for ML techniques [42].

Using deep learning, image processing algorithms were developed that clearly outperformed conventional methods. In October 2015, for the first time, a computer programme could beat a human expert in the highly cognitively demanding game of Go [64, 92]. Autonomous driving would not be possible without deep learning-based image recognition [33]. Typical deep learning neural network types are the convolutional neural network or the recurrent neural network.

In a clinical setting, deep learning has already been successfully used in image-based techniques to classify fractures, osteoarthritis, bone age, tendon tears (reviewed by [4] or to analyse the alignment of the spine [103] or the lower extremity [70]. In addition, decoding lower-limb kinematic parameters was demonstrated using deep learning approaches [9, 27, 65]. Even automated recognition of bone metastases on bone scintigrams has been described [61]. An example of how an algorithm architecture to detect bone metastases on radiographs of the knee is shown in Fig. 4. It is worth noting that deep learning can also affect medical disciplines outside the purely medical scope, such as healthcare facility management [69]. Deep learning has thus the potential to be one of the most transformative technologies to impact orthopaedic surgery. However, for this to happen, the clinical knowledge necessary to identify orthopaedic problems and the technical expertise needed to implement deep learning-based solutions must come together [79].

## Discussion

Although medially omnipresent, AI and ML are probably still a black box to many orthopaedic and trauma specialists. This review aims to provide a basic understanding of what these techniques encompass, how they work, and how they may be used in orthopaedics and traumatology. Understanding the underlying concepts of AI and ML will help to better understand their limitations and imagine their potential.

Over the last few years, ML has become a very popular method to analyse large data sets. Its subdiscipline, deep learning, is now increasingly being used. While deep learning neural networks are difficult to set up, their power outperforms by far that of conventional ML [2].

ML techniques offer the window for a new set of knowledge. Learning from big data allows us to recognise relationships and associations that are impossible to approach with conventional data curation. While classical statistical methods focus on inference, machine learning allows conclusions about both inference and prediction [14]. Automation through ML can cut down on required staff, which is critical in an ageing Western society. Although validation between different institutions and even countries would still be required, such ML models can principally be implemented globally. Although there is a huge body of literature emerging in scientific databases presently, many of the presented models are still far from a quality that would justify their implementation in everyday practice. To date, ML models are particularly effective when being used as clinical decision support systems instead of being used as stand-alone solutions [96]. In case that such models meet, however, the needed criteria of validity, reliability and effectivity, their economic impact would be of relevance both on a microeconomic and a macroeconomic level. Therefore, although initial setup may be associated with financial investment, it can eventually reduce local and global costs [53].

ML programmes are still generally task-specific and thus limited in their flexibility. Within their designated task, ML more and more achieves comparable results as humans or even outperforms them through higher precision, reliability and reduced error rate [58, 98, 99]. This is also due to the fact, that ML algorithms continuously improve by further data input [47]. This will eventually lead humanity to a place that is hard to foretell. At least from a human brain's perspective, the calculation power is almost unlimited in computers. The speed with which tasks can be performed and decisions can be reached is often in the order of magnitude from milliseconds to seconds. While this is generally a convenient feature, this speed is especially advantageous in the trauma and emergency setting, where many decisions are time-critical.

Next to all these powerful strengths of ML, several weaknesses and limitations still lie in that technology. Many supervised learning studies in the literature were, for example, purely conducted retrospectively [25, 28, 49, 52, 97, 106]. This means the outcome was already transparent and independent of the ML calculation. This represents a very valid approach when trying to figure out a meaningful ML technique to predict future cases. One has to take into consideration, however, that such a study design makes these created ML algorithms more prone to hidden or intentional biases [26]. Clinicians require, however, transparent and explainable results to trust and integrate AI-driven recommendations into decision-making processes. Future research should, therefore, give more importance to testing ML systems prospectively. As stated above, ML algorithms can continuously improve [47] with more and more data being fed to the system. Especially in newly developed knowledge areas, such sufficiently large data sets might, however, simply not be available. Sometimes, these data sets take years to implement before an ML algorithm can even start, as is the case for arthroplasty registries.

One weakness of ML tools is that their faulty diagnoses can be challenging to detect and correct as this usually requires going through the entire decision-making process [26, 83]. It may be misleading, to accept recommendations from a deep-learning decision if the role of different factors influencing the model’s decision is unknown or not evaluated. In a recent study, [5], reported that their trained programme to recognise hip fractures on conventional radiographs only reached reasonable results when taking into consideration non-imaging patient factors. When having to base the decision on X-rays alone, the model performed at random, highlighting a rather questionable role of context factors in the decision-making process. The authors conclude that, if computer algorithms inexplicably leverage patient and process variables in their predictions, it remains unclear how doctors should interpret such predictions in the context of other known patient data [5]. To obtain a good ML programme, extensive feeding of the system and continuous input of newly acquired data are thus necessary. ML can only deal with situations it has been trained for. It, therefore, needs to be borne in mind that it can only address statistical rather than literal truths [26, 83].

For many questions addressed by ML, especially with interval-scaled data, other techniques can also objectify the targeted outcome. This means that the performance of the algorithm can be objectively quantified, such as the size of a tumour or the blood flow in a vessel. In medicine, many questions are, however, more complex. This is especially the case when a dichotomous parameter is allocated for a condition that biologically is most likely a continuous variable, such as the presence of rheumatoid arthritis. In mild cases, it is often not clear if the patient has rheumatoid arthritis or not. The decision as to whether a specific patient has rheumatoid arthritis or not is then based on a personal judgement considering various criteria [50]. There are, however, no objective means to verify this decision by some other independent technique. In these circumstances, an ML tool can, at best, be as good as the human observer due to a lack of an objectifiable ground truth beyond the human judgement. Although an ML algorithm could thus theoretically outperform a human in such decisions, it will be hard to actively program it to that end. Even in case of an ML model superiority, the lack of a clear ground truth makes validation of the success of such a model a major scientific challenge. For a summary of the strengths and weaknesses of ML, see also Table 2. Depending on the local background, regulatory and ethical issues, such as patient privacy, informed consent, and a need for adequate validation of AI tools, can make the integration of AI into existing healthcare infrastructures even more complicated.

**Table 2:** Strengths and weaknesses of machine learning

Strengths	Weaknesses
Automation of tasks is possible	ML is task-specific
Can reduce work-load, staff and cost	High implementation and maintenance costs
Once established and validated, model is globally useable	Can only be used in fields, where sufficiently large data sets are available for training
Can provide real-time feedback and fast decisions	Quality of the ML product is highly depended on the quality and quantity of available data
High precision, reliability and low error rate for simple tasks such as object recognition when adequately trained	Prone to faulty diagnosis. ML is useful when results are double checked with expert
Recognises relationships in large data sets that are impossible to grasp with conventional statistical means	Prone to systemic bias given the choice of data or algorithm ("subgroups"with little data not adequately represented)
Model improves over time	“Black box problem”: ML model does not provide information how predications were achieved
	Generates only reliable predictions if the situations concerned are present in the training data

Overcoming the challenges of using AI and machine learning in musculoskeletal medicine requires a multifaceted approach. First, improving data quality is essential for training more accurate models. This can be achieved by ensuring large, diverse, and representative well-annotated data sets that reflect the full spectrum of musculoskeletal conditions. Efforts to standardise data collection methods and address issues of data privacy and security will help to reduce biases and inaccuracies.

Second, developing interpretable AI models can foster greater clinician trust by making model predictions more transparent and understandable. Collaborative efforts between AI developers, clinicians, and regulatory bodies can facilitate the creation of standards for AI tool validation, ensuring their reliability and safety before clinical use. Finally, by including user-friendly interfaces and involving clinicians in the development process, we can ensure that AI tools align with real-world clinical practices.

### Future developments

Presently, it is still necessary to have a human gatekeeper supervising the development of algorithm improvements [104]. This can also be recommended to minimise the risk of automated biases arising from the data. Whether this gatekeeper function can and/or shall be eliminated in the future is not just a technical, but also a philosophical question.

One of the critical capabilities of ML is to find trends and predict future tendencies by applying large data sets [14]. Looking back on revolutionary medical advances over the past decades, excellent improvement potential is presently seen in further individualising treatment strategies. An interesting conundrum is how to bridge the gap between highly personalised medicine on the one hand and the generalising directions given by ML techniques. Despite the increasing availability of massive data sets, the predictive power of most of the available disease models still needs to meet the requirements for clinical practice. Predictive disease models must cover all relevant biotic and abiotic mechanisms driving disease progression in individual patients [32]. An exciting solution could be provided by so-called hybrid models that offer an integrative approach by combining a validated mechanical model with a data-driven ML model [32].

Presently, ML can only partly replace human intelligence in medicine. A dual complementary function is conceivable for numerous applications [16], as it has been used for electrocardiograms for decades [86]. The first analysis is done by the AI, which is then double-checked and verified by a human observer. This step will often remain necessary, given the susceptibility of ML to error. Freeing up humans, however, from one or two critical tasks within a complex process already improves the total outcome, since humans can then focus better on more vital tasks [26].

Concerning future developments in the field, we may expect a deeper market penetration of commercially available tools and devices based on machine learning. This includes the further propagation of robotics in surgery, notably arthroplasty and spine surgery [35], augmented reality in tumour or reconstructive surgery [13, 71], assisted imaging analyses and increasing vocal interfaces with computers through enhanced NLP. We expect that personalised AI-supported musculoskeletal medicine will allow to track specific disease conditions over time, allowing early intervention and proactive management. In addition, patient-specific anatomy will increasingly be integrated into virtual models [102], thus improving pre-operative planning and execution. Employing AI-driven rehabilitation tools or robotics-assisted rehabilitation will also allow a more personalised recovery regime and thus individualised treatment plans. For a broader scope on the future importance and implications of AI in healthcare in general, please see [12].

At least for the next decade, these changes will most likely be linear, which means that they may also be extrapolated from our present standpoint. The significant and unknown Jack-in-the-box in ML is presently the potential development of quantum machine learning. Presently, these computers still need to be readily available on the market. Given the high maintenance requirements, high costs and lack of standardised operation systems, quantum computing is also not ready to be accessible to"normal research". First companies have begun, however, to offer opportunities to use cloud-based quantum computing (e.g., Google Quantum AI, Amazon Braket, IBM Quantum Qiskit, Microsoft Azure). Quantum computing provides exponentially more storage and processing power than binary bit-based computing technology. Numerous applications are conceivable in life sciences, including orthopaedics and traumatology [21, 84]. Where this development will take us is presently hard to fathom. The potential seems enormous. To date ML models based on binary computation can be worse, equally good, or better than humans in their task-specific performance—depending on the preceding training, the quality and quantity of the data and the underlying algorithm.

## Conclusion

ML and its subset deep learning have seen dazzling improvements over the last decade. ML offers powerful tools now suitable for numerous applications to be implemented in clinical use. While the initial setup costs are high, these investments will likely pay off by reducing workload and cost. Until good analyses and predictions are obtained by ML, patience in training and suitable data sets are required. Joint efforts should be undertaken to standardise data collection and data set annotation techniques. Collaborative efforts between AI developers, clinicians, and regulatory bodies could facilitate the creation of standards for AI tool validation, ensuring their reliability and safety before clinical use. Finally, by including user-friendly interfaces and involving clinicians in the development process, we can ensure that AI tools align with real-world clinical practices.

Knowing the strengths and weaknesses of ML will help to put this technique to good use wisely. For the next decade, in a clinical setting especially, a complementary function to human tasks can be expected. Where this journey will further take us is still hard to say. A good ML tool might help to predict it.

## Data Availability

No datasets were generated or analysed during the current study.

## Abbreviations

ML: Machine learning
AI: Artificial intelligence
TKA: Total knee arthroplasty
NLP: Natural language processing
